# A workforce survey of Australian osteopathy: analysis of a nationally-representative sample of osteopaths from the Osteopathy Research and Innovation Network (ORION) project

**DOI:** 10.1186/s12913-018-3158-y

**Published:** 2018-05-10

**Authors:** Jon Adams, David Sibbritt, Amie Steel, Wenbo Peng

**Affiliations:** Australian Research Centre in Complementary and Integrative Medicine (ARCCIM), Faculty of Health, University of Technology Sydney, Level 8, Building 10, 235-253 Jones St, Sydney, NSW 2007 Australia

**Keywords:** Osteopath, Osteopathy, Workforce, Practice-based research network

## Abstract

**Background:**

Limited information is available regarding the profile and clinical practice characteristics of the osteopathy workforce in Australia. This paper reports such information by analysing data from a nationally-representative sample of Australian osteopaths.

**Methods:**

Data was obtained from a workforce survey of Australian osteopathy, investigating the characteristics of the practitioner, their practice, clinical management features and perceptions regarding research. The survey questionnaire was distributed to all registered osteopaths across Australia in 2016 as part of the Osteopathy Research and Innovation Network (ORION) project.

**Results:**

A total of 992 Australian osteopaths participated in this study representing a response rate of 49.1%. The average age of the participants was 38.0 years with 58.1% being female and the majority holding a Bachelor or higher degree qualification related to the osteopathy professional. Approximately 80.0% of the osteopaths were practicing in an urban area, with most osteopaths working in multi-practitioner locations, having referral relationships with a range of health care practitioners, managing patients a number of musculoskeletal disorders, and providing multi-model treatment options.

**Conclusions:**

A total of 3.9 million patients were estimated to consult with osteopaths every year and an average of approximate 3.0 million hours were spent delivering osteopathy services per year. Further research is required to provide rich, in-depth examination regarding a range of osteopathy workforce issues which will help ensure safe, effective patient care to all receiving and providing treatments as part of the broader Australian health system.

**Electronic supplementary material:**

The online version of this article (10.1186/s12913-018-3158-y) contains supplementary material, which is available to authorized users.

## Background

Osteopathy, with an emphasis on whole person care, is a health care system integrating physical examinations and manipulative treatments for a range of conditions [1–3]. Osteopathy has been developed in more than 50 countries and has been regulated in at least 15 countries, including US, UK, and Australia [4]. Amongst the countries with statutory regulation of osteopathy, there are two professional streams in the field of osteopathy – ‘osteopathic physicians’ in the US exclusively and ‘osteopaths’ in other countries [4]. Osteopathic physicians are qualified physicians with full medical practice rights throughout the US and can perform surgery, while, outside of the US, osteopaths are healthcare providers with practice rights who are not licensed to prescribe medications or perform surgery [5]. As a government registered allied health profession in Australia, most private health insurers provide partial reimbursement for osteopathic services [6]. It is worth noting that Australian osteopaths do provide a service under the government’s Medicare Chronic Disease Management Plan [7]. The Australian osteopathic workforce continues to grow, with 2115 registered practicing osteopaths in 2016, compared to 1601 registered practicing osteopaths in 2012 [8]. National-scale studies have estimated that, within a 12-month period, osteopaths provided 3.1 million patient consultations in Australia in 2005 and 3.4 million patient consultations in the US in 2006 [9, 10].

Previous osteopathy workforce studies have largely examined educational issues [11, 12], treatment efficacy for a number of musculoskeletal conditions [13, 14], and osteopathy use amongst either general populations or clinical subgroups across a range of countries including Australia [10, 15]. In addition to the online health workforce dataset (including osteopathy) (16), only three previous studies have examined the profile of Australian osteopaths [16–18] and unfortunately all have been limited in sample size and/or sample focus. One such study undertaken in 2004 restricted data collection to members of an osteopathy association [16], another 2010 study recruited only 54 osteopaths [17], and a third study conducted in 2012 only reported on the profile of osteopaths exiting practice [18]. As such, there is a significant lack of analyses regarding the profile and clinical practice characteristics of Australian osteopaths.

In direct response, this paper aims to provide an in-depth exploration of the practitioner and practice characteristics as well as the clinical practice management characteristics of Australian osteopaths from a nationally-representative sample recruited as part of a practice-based research network (PBRN) project. A PBRN refers to a collaboration of practitioners and practices together with professional academic institutions to facilitate research studies designed for answering clinical questions and to help translate research findings into daily patient care (https://pbrn.ahrq.gov/). Therefore, results reported here also provide sustainable opportunities for further nested sub-studies focusing upon the efficacy and health services use of osteopathy practices.

## Methods

### Recruitment and distribution

The Osteopathy Research and Innovation Network (ORION) project is the first national PBRN of osteopaths in Australia (http://www.orion-arccim.com/) and the recruitment of this project was conducted from July to December 2016. The ORION recruitment invitation pack included an online practitioner questionnaire (accessed via SurveyGizmo™) as well as a consent form relating to joining the ORION PBRN database.

Osteopathy Australia (OA) is a national professional organization representing more than 85% of registered osteopaths in Australia. The invitation pack was distributed to all registered osteopaths via OA networks for OA members and via osteopathy-related conferences/events and the ORION website for non-OA members. Participants were directed to the ORION website (containing the online practitioner questionnaire) and recruitment emails enclosed embedded links directly to the questionnaire. Several email reminders were circulated following the initial invitation pack. The ORION project is designed and conducted by senior researchers at the Faculty of Health, University of Technology Sydney independent of OA who funded the project. This project has been approved by the UTS Human Ethics Committee (approval # 2014000759).

Participants were invited to complete the online practitioner questionnaire and provide consent to be an ongoing member of the ORION PBRN project. The analyses presented in this article report data gathered from all participants who completed the questionnaire regardless of whether the participant provided consent or did not provide consent to be an ongoing member of the ORION PBRN database.

### Questionnaire

A questionnaire with 27 items was administered to registered osteopaths in Australia (Additional file 1). The questionnaire examined osteopathic practitioner characteristics, practice characteristics, clinical management, and research engagement. Participants were questioned about their age, gender, highest osteopathy professional qualification, years in private osteopathy practice, professional organization membership, and roles they have been involved in as an osteopath in the previous 12 months. With regards to practice characteristics the participants were questioned regarding their average patient care hours per week, average patient visits per week, number of practice locations, other health professionals working in the same practice location, professional referral relationships (sending and receiving referrals separately), State/Territory of practice, practice location (urban, rural, remote), use of diagnostic imaging and reason(s), techniques used to assist in clinical diagnosis, use of electronic records and specified software(s) for electronic records, and frequency of using eHealth system, HICAPs and Medicare Easyclaim. Osteopathic clinical management measures consisted of the frequency of discussion with patients in their care/management plan such as diet/nutrition, physical activity/fitness, and medication, frequency of treating patients with a broad range of conditions (including musculoskeletal disorders and non-musculoskeletal disorders), broader patient subgroups (such as older people, people with sports-related injuries, and people with work-related injuries) of those treated, a wide range of techniques/methods (such as strain, soft tissue, and functional techniques) employed in patient management, and perceptions regarding a variety of osteopathy practice issues (such as prescribing rights, expanded referral rights to medical specialists, and expanded diagnostic imaging rights). In addition, the ORION participants were asked about their perceptions relating to a number of issues around osteopathy research.

### Statistical analyses

All data were imported into the statistical software Stata 14. Variables regarding the frequency of osteopaths’ clinical practice and management (response options: never, rarely, sometimes, often) were re-categorised as ‘often’ and ‘never/rarely/sometimes’. Dichotomous and categorical variables are presented in frequencies and percentages and continuous variables are shown in means and standard deviations.

## Results

According to the national population of practicing osteopaths as registered with the Australian Health Practitioner Regulation Agency (AHPRA) - the sole national agency regulating osteopaths amongst other professions in Australia - there were 2020 registered practicing osteopaths in Australia at the time of the ORION recruitment [8] and 992 osteopaths completed the ORION practitioner questionnaire (response rate: 49.1%). Compared to the data provided by AHPRA, the sample of ORION questionnaire respondents was found to be nationally-representative of the national osteopathy workforce with regards to a number of core characteristics - age (*p* = 0.111), gender (*p* = 0.053) and principal place of practice (*p* = 0.990). As such, the workforce sample reported in this paper and facilitated by the ORION project constitutes a nationally-representative sample of the wider osteopathy profession in Australia. Please note, to identify the representativeness of ACORN data, chi-square tests were employed and statistical significance was set at *p* < 0.05.

### Practitioner characteristics

The average age of participants was 38.0 (SD = 10.9) years, with 58.1% being female. The average number of years in private osteopathy practice was 11.4 (SD = 9.0) years. More than half (68.7%) of the osteopaths held a Masters degree related to the osteopathy profession, while 21.6% of participants held a Bachelor or double Bachelor degree related to the osteopathy profession and only 0.5% had a PhD related to the osteopathy profession. In addition to private practice, the respondents also report being involved in other roles as an osteopath in the last 12 months, including volunteer work (16.0%), clinical supervision (15.1%), university teaching (11.7%), professional organisations (10.8%), and research (5.4%).

### Practice characteristics

The respondents spent an average of 28.3 (SD = 11.8) hours per week on patient care and facilitated an average of 37.0 (SD = 18.2) patient visits per week (only 791 participants provided information on the patient visits per week). Most osteopaths report practicing in one location (65.0%), but of those who practice in more than one location, 85.4% practice in two locations and 12.3% practice in three locations. A total of 829 (83.7%) osteopaths practice in a multi-practitioner location, with 64.8% working with another osteopath, 50.5% working with a massage therapist, 19.5% working with a naturopath, 19.3% working with a psychologist or counsellor, and 19.0% working with an acupuncturist in the same practice location. General practitioners (GPs) are the most common health professional to whom osteopaths send referrals (88.5%) as well as the most common health professional from whom the osteopaths report receiving referrals (89.3%), followed by massage therapists (sending: 67.6%; receiving: 76.0%), another osteopath (sending: 51.0%; receiving: 61.9%) and podiatrists (sending: 65.6%; receiving: 47.5%) (Table 1).

**Table 1:** Practitioners in the same practice location and their professional referral relationships with osteopaths

Health professional	Working in the same practice location	Sending referral(s)	Receiving referral(s)
	*n* (%)	*n* (%)	*n* (%)
Another osteopath	643 (64.8)	506 (51.0)	614 (61.9)
General practitioner	72 (7.3)	878 (88.5)	886 (89.3)
Medical specialist	31 (3.1)	443 (44.7)	237 (23.9)
Podiatrist	147 (14.8)	651 (65.6)	471 (47.5)
Physiotherapist	144 (14.5)	331 (33.4)	266 (26.8)
Exercise physiologist	124 (12.5)	398 (40.1)	258 (26.0)
Occupational therapist	19 (1.9)	106 (10.7)	61 (6.1)
Psychologist/Counsellor	191 (19.3)	349 (35.2)	154 (15.5)
Massage therapist	501 (50.5)	671 (67.6)	754 (76.0)
Acupuncturist	188 (19.0)	451 (45.5)	370 (37.3)
Naturopath	193 (19.5)	477 (48.1)	400 (40.3)
Dietician	72 (7.3)	167 (16.8)	39 (3.9)
Nutritionist	78 (7.9)	129 (13.0)	55 (5.5)
Others	201 (20.3)	148 (14.9)	153 (15.4)

The majority of respondents are working in (State/Territory) Victoria (56.4%), followed by New South Wales (26.5%), Queensland (9.0%), Western Australia (3.2%), Tasmania (2.2%), South Australia (1.8%), Australian Capital Territory (1.7%), and the Northern Territory (0.2%). The majority of osteopaths (81.8%) report practicing in an urban area, with only 18.2% practicing in a rural or remote area. More than half (55.9%) of all the osteopaths ‘sometimes’ or ‘often’ refer their patients for diagnostic imaging, and investigation of suspected diagnosis, potential fractures, and unknown pathologies are the most common reasons for the use of diagnostic imaging. In terms of the techniques used to assist in clinical diagnosis, orthopaedic testing (97.6%) and neurological testing (92.5%) are the most frequent options reported amongst the osteopaths. Electronic patient records are commonly used amongst the participants with 73.2% of all the osteopaths using electronic records for initial history, 76.2% for subsequent patient visits, and 74.1% for examination findings.

### Clinical management

The osteopaths typically discuss a number of topics as part of their care/management plans with the patients (Table 2). The most often discussed topics are physical activity/fitness (89.4%), occupational health and safety (51.2%), and stress management (49.4%). People with sports-related injuries (50.6%), people with work-related injuries (36.2%), and pregnant women (34.7%) are the patient subgroups most often consulting the participants. In terms of conditions presented by patients, musculoskeletal disorders are the most frequently treated by the respondents with low back pain (98.7%), neck pain (98.0%), thoracic pain (91.7%) and headache disorders (90.1%) the most common musculoskeletal conditions presented by patients to the respondents (Table 2).

**Table 2:** Osteopathy clinical management. Numbers indicate the frequencies (percentages) of osteopaths on an often basis

Clinical Management	*n* (%)
Patient care discussion	
Diet/nutrition	375 (37.9)
Smoking/Drugs/Alcohol	179 (18.1)
Physical activity/Fitness	886 (89.4)
Occupational health and safety	506 (51.2)
Pain counselling	264 (26.6)
Stress management	489 (49.4)
Nutritional supplements	252 (25.4)
Medications	391 (39.5)
Patient subgroups	
Children (up to 3 years)	156 (15.8)
Children (4 to 18 years)	270 (27.3)
Older people (65 years and over)	572 (57.7)
Aboriginal and Torres Strait Islander people	7 (0.7)
Pregnant women	344 (34.7)
People with sports-related injuries	501 (50.6)
People with work-related injuries	359 (36.2)
People with traffic-related injuries	141 (14.2)
People receiving post-surgical rehabilitation	79 (8.0)
Non-English speaking ethnic groups	33 (3.3)
Presenting conditions	
Neck pain	971 (98.0)
Thoracic pain	909 (91.7)
Low back pain	977 (98.7)
Hip musculoskeletal disorders	744 (75.2)
Knee musculoskeletal disorders	491 (49.7)
Ankle musculoskeletal disorders	333 (33.7)
Foot musculoskeletal disorders	294 (29.7)
Shoulder musculoskeletal disorders	801 (81.0)
Elbow musculoskeletal disorders	251 (25.5)
Wrist musculoskeletal disorders	188 (19.0)
Hand musculoskeletal disorders	121 (12.3)
Postural disorders (including lordosis, thoracic kyphosis, scoliosis)	675 (68.3)
Degenerative spine conditions (including spondylolisthesis)	599 (60.6)
Headache disorders (including cervicogenic, tension)	892 (90.1)
Migraine disorders	400 (40.5)
Spinal health maintenance or prevention	458 (46.4)
Chronic or persistent pain	630 (63.7)
Tendinopathies	410 (41.5)
Temporomandibular joint disorders	183 (18.5)
Non-musculoskeletal disorder(s)	126 (12.9)

The osteopaths report employing a wide range of techniques and methods in their patient management (Table 3), including soft tissue techniques (85.7%), muscle energy techniques (79.5%), and exercise prescription (74.0%). In terms of the future directions of osteopathy, most of the respondents report they would like to see expanded diagnostic imaging rights (83.0%) and expanded referral rights to a number of specialists including sports medicine specialists (79.8%), orthopaedic surgeons (70.9%), and rheumatologists (63.5%).

**Table 3:** Osteopathy methods. Numbers indicate the frequencies (percentages) of osteopaths on an often basis

Techniques/Methods	*n* (%)
Strain/Counterstain	420 (42.4)
Muscle energy techniques	788 (79.5)
High velocity low amplitude/Spinal manipulation	632 (63.8)
Peripheral joint manipulation	393 (39.7)
Soft tissue	848 (85.7)
Myofascial release	612 (61.8)
Cranial techniques	233 (23.5)
Facilitated positional release	166 (16.8)
Needling techniques (eg. dry needling, acupuncture)	234 (23.6)
Visceral techniques	98 (9.9)
Lymphatic pump	84 (8.5)
Autonomic balancing	157 (15.9)
Biodynamic techniques	155 (15.6)
Functional techniques	270 (27.3)
Balanced ligamentous tension/Ligamentous articular strain	349 (35.2)
Exercise prescription	733 (74.0)
Chapmans reflexes	24 (2.4)
Shockwave therapy	18 (1.8)
Ultrasound therapy	27 (2.7)
TENS or other electrotherapy	19 (1.9)
Instrument-assisted manipulative techniques	2 (0.2)
Instrument-assisted soft tissue mobilisation	12 (1.2)
Trigger point therapy	258 (26.1)
Sports taping	122 (12.3)

### Research impact

A majority (74.9%) of respondents report that evidence from research has a moderate to high impact upon their current osteopathy practice (Table 4). The majority of participants consider osteopathy research as useful in: helping patients understand the benefits of osteopathy for their health (84.1%); helping GPs and other conventional health professionals understand the role of osteopathy in health care (93.9%); and providing scientific evidence for what they do as an osteopath (87.3%).

**Table 4:** Osteopaths’ perceptions of the impact of osteopathy research

Perceptions of osteopathy research	Disagree	Neutral	Agree
	n (%)	n (%)	n (%)
Research is useful to help patients understand the benefits of osteopathy for their health	52 (5.2)	106 (10.7)	834 (84.1)
Research is useful to help GPs and other conventional health professionals understand the role of osteopathy in health care	28 (2.9)	30 (3.2)	894 (93.9)
Research is useful to provide scientific evidence for what I do as an osteopath	41 (4.4)	78 (8.3)	820 (87.3)
Research is irrelevant to the professional development of osteopathy in Australia	807 (86.1)	46 (4.9)	84 (8.9)
Impact of osteopathy research	Not high impact	Moderate impact	High impact
	n (%)	n (%)	n (%)
What impact does evidence from research have on your current practice?	249 (25.1)	504 (50.8)	239 (24.1)

## Discussion

This study provides the first large-scale nationally-representative analyses of the osteopathy workforce in Australia and has identified a number of interesting findings. Our analyses show that only 0.5% of the osteopaths have obtained a PhD degree. Additionally, the majority of participating osteopaths perceive at least a moderate impact of evidence from research on their osteopathy practice, and this rate is much higher than that reported in a US study focusing upon osteopathic physicians in a family medicine program [19]. These practitioners’ opinions are supported by the broader announcements of Osteopathy Australia’s calling for more research on osteopathy in Australia, highlighting the importance of research involvement amongst osteopaths [20]. As such, there is an urgent need to conduct further studies in what the osteopaths perceive as useful research, develop personnel research training within the osteopathy profession, and eventually build a sustainable research culture focused on osteopathy to provide effective and safe patient care.

Australian osteopaths, presented in this study, report an average of 37 patient consultations and/or treatments per week across a broad patient age range. A 2011 government report focusing upon the allied health sector in Australia indicated similar patient volumes amongst osteopaths [21]. Based on the average patient visit identified from our study, it is estimated that the Australian osteopathy workforce currently manages approximately 3.9 million patients per year suggesting osteopaths play an important role in health provision in Australia. In addition, our study found that the average weekly hours worked amongst osteopaths was 28 h, which equates to an estimation of 3.0 million hours per year of patient care. It is difficult to compare the work time and patient volume of Australian osteopaths with osteopathy practitioners working in other countries, due to the different health care coverage in clinical practice between osteopaths and osteopathic physicians [22] as well as the scarce national workforce research in the osteopathy profession [23].

Our study showed that GPs were the health care practitioners with whom osteopaths were more likely to have a professional referral relationship, and the prevalence of osteopaths sending referrals to GPs (88.5%) and receiving referrals from GPs (89.3%) in our study is much higher than that reported in previous studies [17, 24]. Such disparity in findings may be partly explained by the research design and sample size employed across the different studies. More importantly, the higher frequency of referrals to and from GPs by osteopaths in our study may reflect an increased trust between osteopaths and GPs over recent years [25, 26] and/or may be related to the increased access to osteopathy care through the Medicare Chronic Disease Management Plan scheme, thus this may be an area for further investigation.

Our study identifies a larger proportion of osteopaths practicing in urban areas compared to rural and remote areas, which is consistent with latest Australian government statistics [27] and a US research report [28]. In supplement to this finding, Australian middle-aged women residing in regional areas were found to be more likely to utilise osteopathy services compared to those living in urban areas [29]. Also, the rural population in the US is generally older with worse health status and less private insurance coverage compared to its urban counterpart [28]. As such, research is required to further explore the practice of osteopathy across the urban/non-urban divide including an examination of possible competing or complementary motivations and reasons for any differences in such practice.

Osteopaths in our study report discussing a range of health-related topics with their patients as part of osteopathic care including physical activity/fitness, stress management, and occupational health and safety. This diversity of topics discussed by osteopaths with patients supports the findings of a previous study also focusing upon Australian osteopaths [30]. Potentially, it appears that osteopaths are already playing an active part in encouraging public health/lifestyle changes in their patients but perhaps the potential can be harnessed further. Additional details about what information is transferred to patients by osteopaths would be advantageous, as it could provide insights as to the exact role osteopaths play in promoting health. Such information could also assist patients, other health care practitioners and policy-makers in making decisions concerning consultation and/or referral to osteopaths.

The findings of this workforce study are limited by a number of issues. The survey data were self-reported by osteopaths so the findings may be impacted by recall bias. Further, the ORION practitioner questionnaire was broad in coverage and as such the depth of investigation into specific issues was therefore limited.

## Conclusion

Our study provides a first opportunity to explore a range of workforce issues using a large, nationally-representative sample of Australian osteopaths. The Australian osteopathic workforce is generally working in multi-practitioner locations, holding multi-disciplinary practitioner relations, and prescribing multi-model treatment options. However, the research engagement and capacity relating to the Australian osteopathy practice remains limited. There is a need for further studies on osteopathic practice and practitioners in order to understand the full potential of the profession within the Australian health care system and to improve effective, safe and coordinated treatment for patients.

## Additional file

Additional file 1: ORION practitioner questionnaire. A workforce questionnaire on Australian osteopathy. (PDF 322 kb)

## Abbreviations

AHPRA: Australian Health Practitioner Regulation Agency
GP: General practitioner
OA: Osteopathy Australia
ORION: Osteopathy Research and Innovation Network
PBRN: National practice-based research network
