# Supplementary material for: A workforce survey of Australian osteopathy: analysis of a nationally-representative sample of osteopaths from the Osteopathy Research and Innovation Network (ORION) project
Source: BMC Health Serv Res. 2018 May 10;18:352. doi: 10.1186/s12913-018-3158-y (PMC5946419; doi:10.1186/s12913-018-3158-y)
Supplement: Supplementary file 1 — ORION practitioner questionnaire. A workforce questionnaire on Australian osteopathy. (PDF 322 kb) [file 12913_2018_3158_MOESM1_ESM.pdf]

ORION PRACTITIONER QUESTIONNAIRE

Osteopathy practitioner characteristics

1) What is your age in years? ____

2) What is your gender?
( ) Male
( ) Female

3) Are you currently in private osteopathy practice?
( ) No
( ) Yes, how many years: ____

4) What is the highest level of osteopathy professional qualification that you hold?
( ) Diploma
( ) Advanced Diploma
( ) Bachelor (or Double Bachelor) Degree
( ) Masters Degree
( ) PhD
( ) Other (please specify): ____

5) Are you a member of any of the following professional organisations? (select all that apply)
[ ] OA
[ ] CA (formerly COCA)
[ ] SMA
[ ] Other(s) (please specify): ____

6) Indicate all the roles in which you have been involved <u>as an osteopath</u> over the last 12 months: (select all that apply)
[ ] University teaching
[ ] Clinical supervision
[ ] Professional organisations
[ ] Private practice
[ ] Research
[ ] Volunteer work
[ ] Other(s) (please specify): ____

Practice characteristics

7) How many of the following would you provide on average, per week?
a) Patient care hours: ____
b) Patient visits: ____

8) Do you practice in more than one location?
( ) No
( ) Yes (how many in total): ____

9) Do you work with other health professionals in the same practice location(s)?
( ) No
( ) Yes
Please indicate all other health professionals working in your practice location(s): (select all that apply)
[ ] Another Osteopath
[ ] GP
[ ] Medical Specialist
[ ] Podiatrist
[ ] Physiotherapist
[ ] Exercise Physiologist
[ ] Occupational Therapist
[ ] Psychologist/Counsellor
[ ] Massage therapist
[ ] Acupuncturist
[ ] Naturopath
[ ] Dietician
[ ] Nutritionist
[ ] Other(s) (please specify): ____

10) Do you <u>send</u> referrals to other health professionals?
( ) No
( ) Yes
Please indicate all other health professionals you send referrals: (select all that apply)
[ ] Another Osteopath
[ ] GP
[ ] Medical Specialist
[ ] Podiatrist
[ ] Physiotherapist
[ ] Exercise Physiologist
[ ] Occupational Therapist
[ ] Psychologist/Counsellor
[ ] Massage therapist
[ ] Acupuncturist
[ ] Naturopath
[ ] Dietician
[ ] Nutritionist

[ ] Other(s) (please specify): ____

11) Do you <u>receive</u> referrals from other health professionals?
( ) No
( ) Yes
Please indicate all other health professionals you receive referrals: (select all that apply)
[ ] Another Osteopath
[ ] GP
[ ] Medical Specialist
[ ] Podiatrist
[ ] Physiotherapist
[ ] Exercise Physiologist
[ ] Occupational Therapist
[ ] Psychologist/Counsellor
[ ] Massage therapist
[ ] Acupuncturist
[ ] Naturopath
[ ] Dietician
[ ] Nutritionist
[ ] Other(s) (please specify): ____

12) In which State/Territory do you practice? (select all that apply)
[ ] NSW
[ ] VIC
[ ] QLD
[ ] WA
[ ] SA
[ ] TAS
[ ] NT
[ ] ACT

13) Which of the following best describes your practice location(s)? (select all that apply)
[ ] Urban
[ ] Rural
[ ] Remote

14) How frequently do you refer your patients for diagnostic imaging?
( ) Never
( ) Rarely
( ) Sometimes
( ) Often

15) For what reason(s) do you refer your patients for diagnostic imaging? (select all that apply)
[ ] Investigation of unknown pathologies
[ ] Investigation of suspected diagnosis
[ ] Investigation of potential fractures

[ ] Rule out risk factors prior to treatment
[ ] General screening of the spine
[ ] Other(s) (please specify): ____

16) Which of the following techniques do you use to assist in clinical diagnosis? (select all that apply)
[ ] Orthopaedic testing
[ ] Clinical assessment algorithm
[ ] Neurological testing
[ ] Screening questionnaire
[ ] Cranial nerve testing
[ ] Other(s) (please specify): ____

17) Indicate when you use electronic records: (select all that apply)
[ ] Initial history
[ ] Examination findings
[ ] Subsequent patient visits
[ ] Never
If you do use electronic records, what software do you use? (select all that apply)
[ ] Cliniko
[ ] Mindbody
[ ] Front desk
[ ] eAlth
[ ] TM2
[ ] Other(s) (please specify): ____

18) How frequently do you share your patient records with the government's eHealth system?
( ) Never
( ) Rarely
( ) Sometimes
( ) Often

19) How frequently do you use a HICAPS terminal?
( ) Never
( ) Rarely
( ) Sometimes
( ) Often

20) How frequently do you use Medicare Easyclaim for chronic disease management patients?
( ) Never
( ) Rarely
( ) Sometimes
( ) Often

Clinical management

21) How frequently do you discuss the following in your care/management plans?

	Never	Rarely	Sometimes	Often
Diet/Nutrition	( )	( )	( )	( )
Smoking/Drugs/Alcohol	( )	( )	( )	( )
Physical activity/Fitness	( )	( )	( )	( )
Occupational health and safety	( )	( )	( )	( )
Pain counselling	( )	( )	( )	( )
Stress management	( )	( )	( )	( )
Nutritional supplements (including vitamins, minerals, herbs)	( )	( )	( )	( )
Medications (including for pain/inflammation)	( )	( )	( )	( )
Other(s)	( )	( )	( )	( )

Please specify the "other(s)": ______________________________

22) How frequently do you treat patients that present with the following conditions?

	Never	Rarely	Sometimes	Often
Neck pain	( )	( )	( )	( )
Thoracic pain	( )	( )	( )	( )
Low back pain	( )	( )	( )	( )
Hip musculoskeletal disorders	( )	( )	( )	( )
Knee musculoskeletal disorders	( )	( )	( )	( )
Ankle musculoskeletal disorders	( )	( )	( )	( )
Foot musculoskeletal disorders	( )	( )	( )	( )
Shoulder musculoskeletal disorders	( )	( )	( )	( )
Elbow musculoskeletal disorders	( )	( )	( )	( )
Wrist musculoskeletal disorders	( )	( )	( )	( )
Hand musculoskeletal disorders	( )	( )	( )	( )
Postural disorders (including lordosis, thoracic kyphosis, scoliosis)	( )	( )	( )	( )
Degenerative spine conditions (including spondylolisthesis)	( )	( )	( )	( )
Headache disorders (including cervicogenic, tension)	( )	( )	( )	( )

Migraine disorders	( )	( )	( )	( )
Spinal health maintenance or prevention	( )	( )	( )	( )
Chronic or persistent pain	( )	( )	( )	( )
Tendinopathies	( )	( )	( )	( )
Temporomandibular joint (TMJ) disorders	( )	( )	( )	( )
Non-musculoskeletal disorder(s)	( )	( )	( )	( )
Other	( )	( )	( )	( )

Please specify the "non-musculoskeletal disorder(s)":

____

Please specify the "other":

____

23) How frequently do you treat the following patient subgroups?

	**Never**	**Rarely**	**Sometimes**	**Often**
Children (up to 3 years)	( )	( )	( )	( )
Children (4 to 18 years)	( )	( )	( )	( )
Older people (65 years or over)	( )	( )	( )	( )
Aboriginal and Torres Strait Islander people	( )	( )	( )	( )
Pregnant women	( )	( )	( )	( )
People with sports-related injuries	( )	( )	( )	( )
People with work-related injuries (covered by Workers' Compensation Scheme)	( )	( )	( )	( )
People with work-related injuries (***not*** covered by Workers' Compensation Scheme)	( )	( )	( )	( )
People with traffic-related injuries (covered by MAA/SIRA, TAC, MAIC, MAC, ICWA, MAIB, TIO)	( )	( )	( )	( )
People with traffic-related injuries (***not*** covered by MAA/SIRA, TAC, MAIC, MAC, ICWA, MAIB, TIO)	( )	( )	( )	( )
People receiving post-surgical rehabilitation	( )	( )	( )	( )
Non-English speaking ethnic group(s)	( )	( )	( )	( )

Other	( )	( )	( )	( )

Please specify the "non-English speaking ethnic group(s)":

____

Please specify the "other":

____

24) How frequently do you employ the following Techniques/Methods in your patient management?

	Never	Rarely	Sometimes	Often
Strain/Counterstrain	( )	( )	( )	( )
Muscle energy techniques	( )	( )	( )	( )
HVLA (high velocity low amplitude)/Spinal manipulation	( )	( )	( )	( )
Peripheral joint manipulation	( )	( )	( )	( )
Soft tissue	( )	( )	( )	( )
Myofascial release	( )	( )	( )	( )
Cranial techniques	( )	( )	( )	( )
Facilitated positional release	( )	( )	( )	( )
Needling techniques (eg. dry needling, acupuncture)	( )	( )	( )	( )
Visceral techniques	( )	( )	( )	( )
Lymphatic pump	( )	( )	( )	( )
Autonomic balancing	( )	( )	( )	( )
Biodynamic techniques	( )	( )	( )	( )
Functional techniques	( )	( )	( )	( )
Balanced ligamentous tension/Ligamentous articular strain	( )	( )	( )	( )
Exercise prescription	( )	( )	( )	( )
Chapmans reflexes	( )	( )	( )	( )
Shockwave therapy	( )	( )	( )	( )
Ultrasound therapy	( )	( )	( )	( )
TENS or other electrotherapy	( )	( )	( )	( )
Instrument-assisted manipulative techniques	( )	( )	( )	( )
Instrument-assisted soft tissue mobilisation	( )	( )	( )	( )

Trigger point therapy	( )	( )	( )	( )
Sports taping	( )	( )	( )	( )
Other technique(s)/Method(s)	( )	( )	( )	( )

Please specify the "other technique(s)/method(s)":

____

25) Do you think osteopathy practice should include the following aspects in the future?

	No	Unsure	Maybe	Definitely
Prescribing rights	( )	( )	( )	( )
Expanded referral rights to an Orthopaedic Surgeon	( )	( )	( )	( )
Expanded referral rights to a Paediatrician	( )	( )	( )	( )
Expanded referral rights to a Sports Medicine Specialist	( )	( )	( )	( )
Expanded referral rights to a Rheumatologist	( )	( )	( )	( )
Expanded referral rights to other Medical Specialists	( )	( )	( )	( )
Expanded diagnostic imaging rights	( )	( )	( )	( )

Research

26) To what extent do you agree or disagree with the following statements?

a) Research is useful to help patients understand the benefits of osteopathy for their health.
( ) Strongly Disagree
( ) Disagree
( ) Neutral
( ) Agree
( ) Strongly Agree

b) Research is useful to help GPs and other conventional health professionals understand the role of osteopathy in health care.
( ) Strongly Disagree
( ) Disagree
( ) Neutral
( ) Agree
( ) Strongly Agree

c) Research is useful to provide scientific evidence for what I do as an osteopath.
( ) Strongly Disagree
( ) Disagree
( ) Neutral
( ) Agree
( ) Strongly Agree

d) Research is irrelevant to the professional development of osteopathy in Australia.
( ) Strongly Disagree
( ) Disagree
( ) Neutral
( ) Agree
( ) Strongly Agree

27) What impact does evidence from research have on your current practice?
( ) No impact at all
( ) Minimal impact
( ) Moderate impact
( ) High impact
